# A Case of Papillary Fibroelastoma of the Aortic Valve Causing an Embolic Ischemic Stroke

**DOI:** 10.7759/cureus.28208

**Published:** 2022-08-20

**Authors:** Rafsan Ahmed, Amirhossein Moaddab, Suzette Graham-Hill

**Affiliations:** 1 Department of Internal Medicine, State University of New York (SUNY) Downstate Health Sciences University, New York City, USA; 2 Department of Cardiology, State University of New York (SUNY) Downstate Health Sciences University, New York City, USA; 3 Department of Cardiology, Kings County Hospital Center, New York City, USA

**Keywords:** transesophageal echocardiography, transthoracic echocardiography, stroke, primary cardiac tumor, papillary fibroelastoma

## Abstract

Papillary fibroelastomas (PFEs) are the second most common primary cardiac tumors after myxomas. They are typically located on the aortic valve and comprise a short pedicle with multiple papillary fronds. PFEs are benign but highly friable in nature. Patients can be asymptomatic or present with severe thromboembolic complications. Echocardiography is the modality of choice for the diagnosis of these masses and surgical resection is indicated even in asymptomatic patients. Here, we have presented a case of a 53-year-old male who presented with a stroke after embolization of a PFE.

## Introduction

Cardiac tumors are rare entities that encompass a broad set of masses. The overall prevalence of primary cardiac tumors ranges from 0.0017% to 0.28%, and papillary fibroelastomas (PFEs) are the second most common benign cardiac neoplasms after myxomas [1,2]. Clinically, patients can be asymptomatic or present with severe thromboembolic complications, myocardial ischemia, heart failure, syncope, infarction, or stroke [3,4]. Here, we have presented a case of a 53-year-old male with no past medical history who presented with a large left posterior cerebral artery (PCA) stroke as a result of embolization from a PFE.

## Case presentation

**Figure 1:**
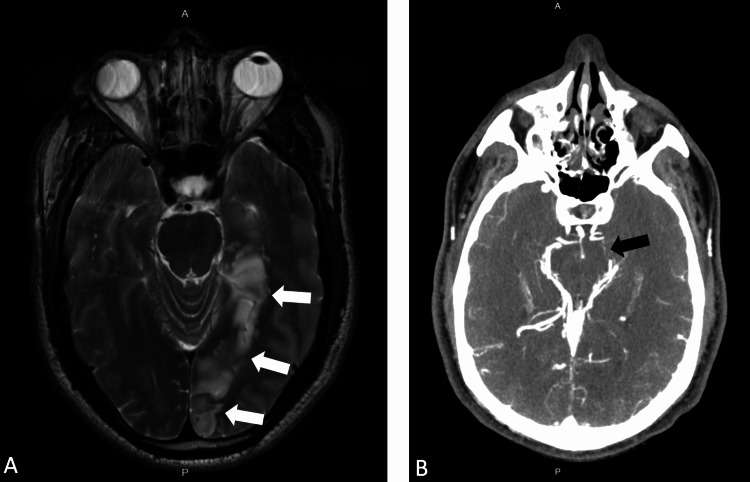
(A) Axial section of the brain MRI scan showing a large, acute left PCA territory infarction (white arrows) involving the left occipital, medial temporal, and parietal lobes, the corpus callosum, and the left thalamus. (B) CT angiogram of the head showing occlusion of the proximal P2 segment (black arrow) of the left PCA A: anterior, P: posterior, MRI: magnetic resonance imaging, CT: computed tomography, PCA: posterior cerebral artery

**Figure 2:**
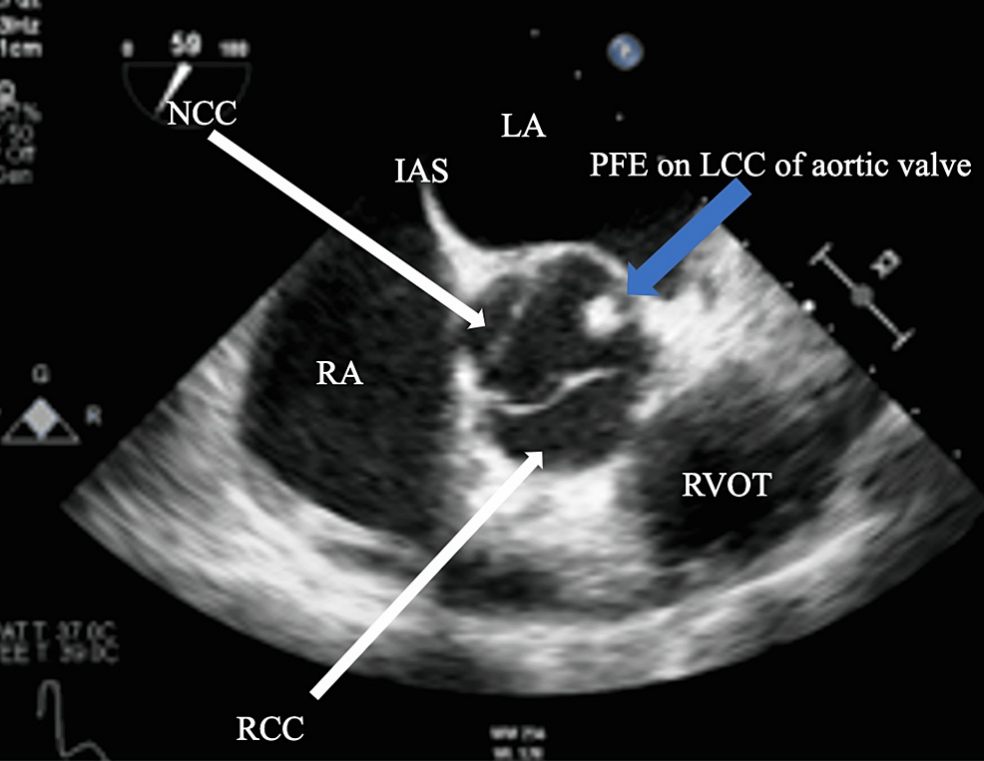
Midesophageal view of the TEE showing the papillary fibroelastoma on the left coronary cusp of the aortic valve (blue arrow) RA: right atrium, LA: left atrium, RVOT: right ventricular outflow tract, IAS: inter atrial septum, RCC: right coronary cusp, LCC: left coronary cusp, NCC: non-coronary cusp, PFE: papillary fibroelastoma, TEE: transesophageal echocardiogram

**Figure 3:**
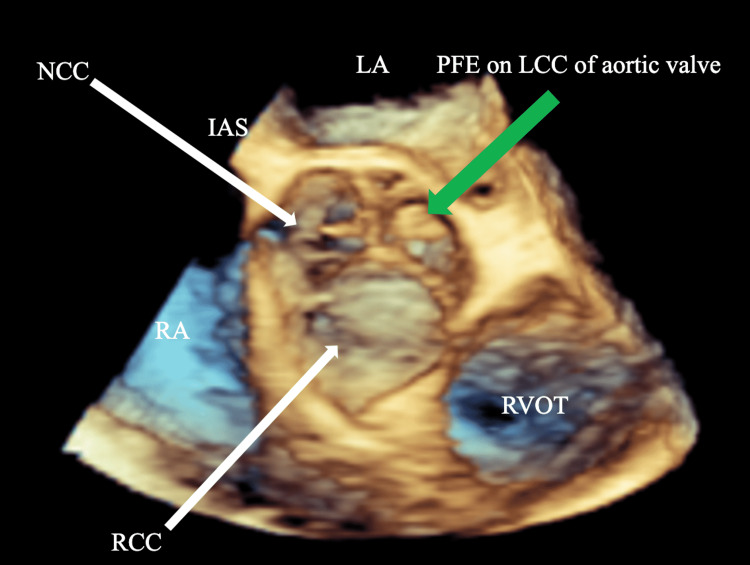
Three-dimensional midesophageal view of the TEE showing the papillary fibroelastoma on the left coronary cusp of the aortic valve (green arrow) RA: right atrium, LA: left atrium, RVOT: right ventricular outflow tract, IAS: inter-atrial septum, RCC: right coronary cusp, LCC: left coronary cusp, NCC: non-coronary cusp, PFE: papillary fibroelastoma, TEE: transesophageal echocardiogram

A 53-year-old male with no previously diagnosed medical conditions presented to the emergency department with left-sided occipital numbness, impaired vision in the right eye, as well as left thigh numbness. Magnetic resonance imaging (MRI) of the brain demonstrated the presence of a large, acute left PCA territory infarction involving the left occipital, medial temporal, and parietal lobes, the corpus callosum, and the left thalamus (Figure 1A). Computed tomography (CT) angiogram of the head was significant for an occlusion of the proximal P2 segment of the left PCA (Figure 1B). The patient subsequently underwent a transthoracic echocardiogram (TTE) that showed moderate concentric left ventricular hypertrophy, a left ventricular ejection fraction of 55%-60%, and dilated left atrium (diameter 5.3 cm). Treatment was started with dual-antiplatelet and high-intensity statin therapy. He was discharged with ambulatory cardiac monitoring and underwent a transesophageal echocardiogram (TEE) that showed no evidence of thrombus in the left atrial appendage. Also, the bubble study did not show any evidence of atrial septal defect or patent foramen ovale. However, an echogenic mass (0.6 x 0.6 cm) on the left coronary cusp of the the aortic valve was seen suggestive of a PFE (Figures 2, 3). Ambulatory cardiac monitoring for 14 weeks did not show any evidence of atrial fibrillation or atrial flutter. The patient was referred for surgery and after successful surgical resection, PFE was confirmed on pathology.

## Discussion

More than 95% of PFEs originate from the left side of the heart, and risk factors for their development remain unclear. Most PFEs are incidentally found in patients with an average age of 60 years. PFEs have an appearance like that of a sea anemone with a short pedicle and multiple papillary fronds. They can range from 2 to 70 mm in size with a mean of 9 mm [3,4]. Histologically, they consist of avascular papillary tissue surrounded by an endothelial layer [5]. The majority of PFEs (>80%) originate from valvular surfaces: 36% on the aortic valve, 29% on the mitral valve, 11% on the tricuspid valve, and 7% on the pulmonary valves. The remaining 20% occur on extra-valvular surfaces of the heart including the left ventricle, left atrium, left atrial appendage, eustachian valve, and right ventricle, in the order of decreasing frequency [3,4]. In very rare cases, PFEs can arise from the wall of the aorta [6].

TTE and TEE are the most used diagnostic modalities for cardiac masses. TTE has a sensitivity and specificity of 88.8% and 88.7%, respectively for PFEs. For PFEs <0.2 cm, TEE sensitivity compared to TTE is 76.6% vs. 61.9%, respectively [4]. Echocardiograms show PFEs as mobile, pedunculated or sessile masses, either attached to the valvular structure or the endocardial surface, that often prolapse into the cardiac chambers during systole or diastole [3,4]. They may appear speckled with echolucencies and surrounded by a stippled pattern representing the papillary fronds [3]. Recently, three-dimensional echocardiography has been used for the better delineation of attachment points and relation with surrounding structures that is useful in determining the valvular function after surgical resection [7]. Although echocardiography remains the primary diagnostic modality for intracardiac masses, cardiac CT and MR are playing an increasingly important role as they allow for the evaluation of extracardiac extent of the disease [8].

Presently, there are no treatment guidelines for PFE. In patients with embolic events, surgical resection is strongly recommended. PFEs are resected with associated endocardial tissue and special care is taken to avoid fragmentation. Surgical resection is curative, safe and well tolerated. Intraoperative TEE is done to assess the valvular function and long-term TEE follow-up studies are done to confirm the absence of re-growth [9,10].

## Conclusions

PFEs are the second most common benign cardiac neoplasms after myxomas. Transthoracic and transesophageal echocardiography is essential in their diagnosis. Although of benign nature, PFEs have a high risk of thromboembolic complications due to their friable nature; as a result, surgical resection is strongly recommended in patients with embolic events. Surgical management is safe and is usually curative.
